# Abemaciclib, A Selective CDK4/6 Inhibitor, Restricts the Growth of Pediatric Ependymomas

**DOI:** 10.3390/cancers12123597

**Published:** 2020-12-01

**Authors:** Muh-Lii Liang, Chun-Han Chen, Yun-Ru Liu, Man-Hsu Huang, Yu-Chen Lin, Tai-Tong Wong, Sey-En Lin, Shing-Shiung Chu, Yi-Huei Ding, Tsung-Han Hsieh

**Affiliations:** 1 Department of Neurosurgery, Mackay Memorial Hospital, Taipei 104, Taiwan; liang4617@hotmail.com (M.-L.L.); dingmeatbun@gmail.com (Y.-H.D.); 2 Department of Medicine, Mackay Medical College, New Taipei City 252, Taiwan; 3 Department of Pharmacology, School of Medicine, College of Medicine, Taipei Medical University, Taipei 110, Taiwan; brianchc@tmu.edu.tw (C.-H.C.); amylin0083@tmu.edu.tw (Y.-C.L.); 4 Cell Physiology and Molecular Image Research Center, Wan Fang Hospital, Taipei Medical University, Taipei 116, Taiwan; 5 Graduate Institute of Medical Sciences, College of Medicine, Taipei Medical University, Taipei 110, Taiwan; 6 Joint Biobank, Office of Human Research, Taipei Medical University, Taipei 110, Taiwan; d90444002@tmu.edu.tw; 7 Department of Pathology, Shuang-Ho Hospital, Taipei Medical University, New Taipei City 235, Taiwan; 19225@s.tmu.edu.tw; 8 Institutes of Clinical Medicine, College of Medicine, Taipei Medical University, Taipei 110, Taiwan; ttwong99@gmail.com; 9 Division of Pediatric Neurosurgery, Department of Neurosurgery, Taipei Medical University Hospital, Taipei Medical University, Taipei 110, Taiwan; dog52037@gmail.com; 10 Neuroscience Research Center, Taipei Medical University Hospital, Taipei 110, Taiwan; 11 Pediatric Brain Tumor Program, Taipei Cancer Center, Taipei Medical University, Taipei 110, Taiwan; 12 Department of Anatomic Pathology, New Taipei Municipal Tucheng Hospital, Chang Gung Memorial Hospital and Chang Gung University, New Taipei City 236, Taiwan; limxianx@tmu.edu.tw

**Keywords:** pediatric ependymomas, abemaciclib, patient-derived xenografts

## Abstract

**Simple Summary:**

Pediatric ependymomas are malignant pediatric brain tumors, and one-third of patients exhibit recurrence within two years of initial treatment. Therefore, this study aimed to find new agents to overcome these chemoresistant tumors and defer radiotherapy treatment. By using integrated bioinformatics and experimental validation, we demonstrated that abemaciclib, a CDK4/6 inhibitor, effectively inhibited cell proliferation and induced cell death. Therefore, treatment with abemaciclib showed encouraging results in preclinical pediatric ependymoma models and provide a new therapeutic strategy in the future.

**Abstract:**

Pediatric ependymomas are a type of malignant brain tumor that occurs in children. The overall 10-year survival rate has been reported as being 45–75%. Maximal safe surgical resection combined with adjuvant chemoradiation therapy is associated with the highest overall and progression-free survival rates. Despite aggressive treatment, one-third of ependymomas exhibit recurrence within 2 years of initial treatment. Therefore, this study aimed to find new agents to overcome chemoresistance and defer radiotherapy treatment since, in addition, radiation exposure may cause long-term side effects in the developing brains of young children. By using integrated bioinformatics and through experimental validation, we found that at least one of the genes *CCND1* and *CDK4* is overexpressed in ependymomas. The use of abemaciclib, a highly selective CDK4/6 inhibitor, effectively inhibited cell proliferation and reduced the expression of cell-cycle-related and DNA-repair-related gene expression via the suppression of RB phosphorylation, which was determined through RNA-seq and Western blot analyses. Furthermore, abemaciclib effectively induced cell death in vitro. The efficiency of abemaciclib was validated in vivo using subcutaneously implanted ependymoma tissues from patient-derived xenografts (PDXs) in mouse models. Treatment with abemaciclib showed encouraging results in preclinical pediatric ependymoma models and represents a potential therapeutic strategy for treating challenging tumors in children.

## 1. Introduction

In children, ependymomas account for 5% of all pediatric central nervous system tumors [1]. The standard treatment for an ependymoma includes surgery and combined adjuvant radiotherapy. Gross-total resection (GTR) is considered to be the most important factor for overall survival (OS) and progression-free survival (PFS) [2,3]. In our previously published analysis of patient outcomes, gross-total resection was shown to improve 10-year PFS and OS, with adjuvant radiation improving 10-year OS [2]. Furthermore, Pejavar et al. demonstrated that adjuvant radiotherapy effectively improved PFS (*p* = 0.045) [4]. Although adjuvant radiotherapy has benefits in terms of clinical outcomes when used at a young age and for less than three years, its use is controversial because of the long-term sequelae related to intelligence. To delay radiation treatment, conventional or intensive chemotherapy is needed to control disease progression. However, the use of either a single agent or a combination of drugs still has low treatment efficacy in pediatric ependymoma due to chemoresistance [5]. Several studies have brought about some insights into the underlying mechanisms of chemoresistance, including observed overexpression of multidrug resistant protein 1 (MDR1) [6], O6-methylguanine-DNA-methyltransferase (MGMT) [7], and DNA synthesis and repair enzyme [8]. Therefore, the identification of a novel and effective therapy for pediatric ependymoma treatment is urgently needed.

Cell growth is rigorously regulated by the cell cycle. The cell cycle is divided into four stages: G0/G1, S, G2, and M phases. These cell cycle phases are tightly regulated by several cyclin-dependent kinases (CDKs) [9]. CDK4 and CDK6 are highly homologous serine/threonine kinases activated by D-type cyclin (cyclin D1–3). The activation of CDK4/6–cyclin D1 complex was shown to suppress RB activity via phosphorylation, thus facilitating the G1/S transition [9]. In normal cells, CDK4/6 is rigorously regulated by members of the INK4 CDK inhibitor family; however, genetic changes or overexpression of cyclin D1-3, CDK4/6, and INK4 family members cause the cell cycle to become dysregulated, resulting in tumor formation. Studies have shown that amplification or overexpression of cyclin D1 and CDK4/6 occurs in several tumors, such as glioma, melanoma, head and neck cancers, non–small-cell lung cancer, and breast cancer [10,11]. Because CDK4/6 and cyclin D1 are often mutated or overexpressed in tumors, they constitute potential therapeutic targets. In recent years, the Food and Drug Administration (FDA) has approved three CDK4/6 inhibitors, namely palbociclib, ribociclib, and abemaciclib [12], which have been used to treat patients with HR-positive, HER2-negative advanced, and metastatic breast cancers [13,14]. CDK4/6 amplification has been observed in adult glioblastoma, and some studies have demonstrated that CDK4/6 inhibitors prevent cell proliferation and tumor growth in NOD-SCID mouse models [15,16,17]. Furthermore, studies have shown that palbociclib inhibits cell proliferation and tumor growth in some pediatric brain tumors, including atypical teratoid rhabdoid tumors (AT/RTs), medulloblastomas, and ependymomas [2,18,19].

By analyzing differential gene expression signatures between normal and tumor public ependymoma microarray datasets, we identified that most of the cell-cycle-associated genes are upregulated, and CDK4/6-cyclin D1 complexes are the major upstream regulators in the cell cycle process. Based on its greater ability to passively cross the blood–brain barrier (BBB) than palbociclib [20], we selected abemaciclib for treatment of two primary pediatric ependymoma cell lines and demonstrated that abemaciclib could suppress cell proliferation at a lower dosage and caused cell death in ependymoma cells at higher concentrations. Treatment of ependymoma-patient-derived xenograft (PDX) mice with abemaciclib also demonstrated that abemaciclib could suppress tumor growth. Furthermore, high-throughput mRNA-seq showed that abemaciclib treatment inhibited the expression of genes involved in the cell cycle, DNA replication, and DNA repair. In summary, the present study highlights that a dysregulated cell cycle is one of the major pathways leading to the development of pediatric ependymomas and that abemaciclib can induce cell death and inhibit tumor growth. Therefore, the drug provides a chance to alleviate ependymoma progression.

## 2. Results

### 2.1. Exploration of Specific Therapeutic Molecules in Ependymomas

**Figure 1:**
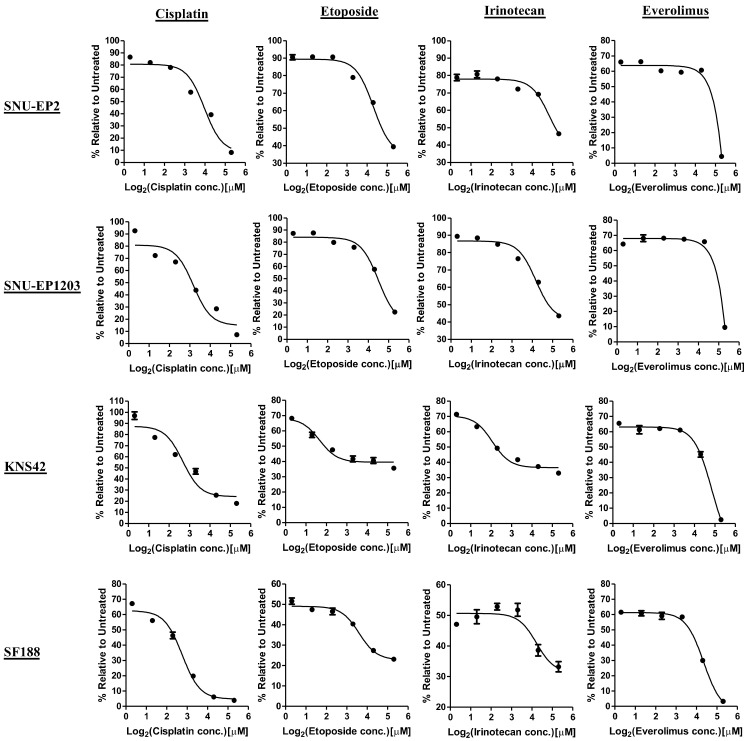
Ependymoma cells are resistant to currently used chemotherapy agents. SNU-EP2, SNU-EP1203, KNS42, and SF188 cells were treated with the indicated concentrations of cisplatin, etoposide, irinotecan, and everolimus. The IC_50_ values were determined by using MTT assay. The IC_50_ value of cisplatin was 14.18 µM in SNU-EP2, 8.67 µM in SNU-EP1203, 8.95 µM in KNS42, and 4.07 µM in SF188. The IC_50_ value of etoposide was 31.61 µM in SNU-EP2, 24.38 µM in SNU-EP1203, 4.39 µM in KNS42, and 1.74 M in SF188. The IC_50_ value of irinotecan was 36.92 µM in SNU-EP2, 33.37 µM in SNU-EP1203, 4.87 µM in KNS42, and 11.38 µM in SF188. The IC_50_ value of everolimus was 23.8 µM in SNU-EP2, 25.62 µM in SNU-EP1203, 16.99 µM in KNS42, and 12.98 µM in SF188. Data are represented as the mean ± SD of triplicate wells and are representative of two independent experiments.

A previous study demonstrated that ependymomas are chemoresistant tumors [5]. Thus, we first evaluated whether our two primary pediatric ependymoma cell lines, SNU-EP2 and SNU-EP1203, were resistant to the current chemotherapeutic agents using MTT assay. SNU-EP2, SNU-EP1203, and two types of pediatric glioblastoma cells, SF188 and KNS42, were treated with cisplatin, etoposide, irinotecan, and everolimus in serial concentrations for 72 h. The average IC_50_ values of cisplatin, etoposide, irinotecan, and everolimus were 11.43 ± 3.9, 28 ± 5.11, 35.15 ± 2.51, and 24.71 ± 1.29 µM, respectively, for the ependymoma cells (Figure 1). By contrast, these four drugs displayed higher levels of potency in glioblastoma cells, where the average IC_50_ values were 6.51 ± 3.45, 3.07 ± 1.87, 8.13 ± 4.6, and 14.99 ± 2.84 µM, respectively (Figure 1). These results suggest that the SNU-EP2 and SNU-EP1203 ependymoma cells are more resistant to chemotherapeutic agents than pediatric glioblastoma cells.

**Figure 2:**
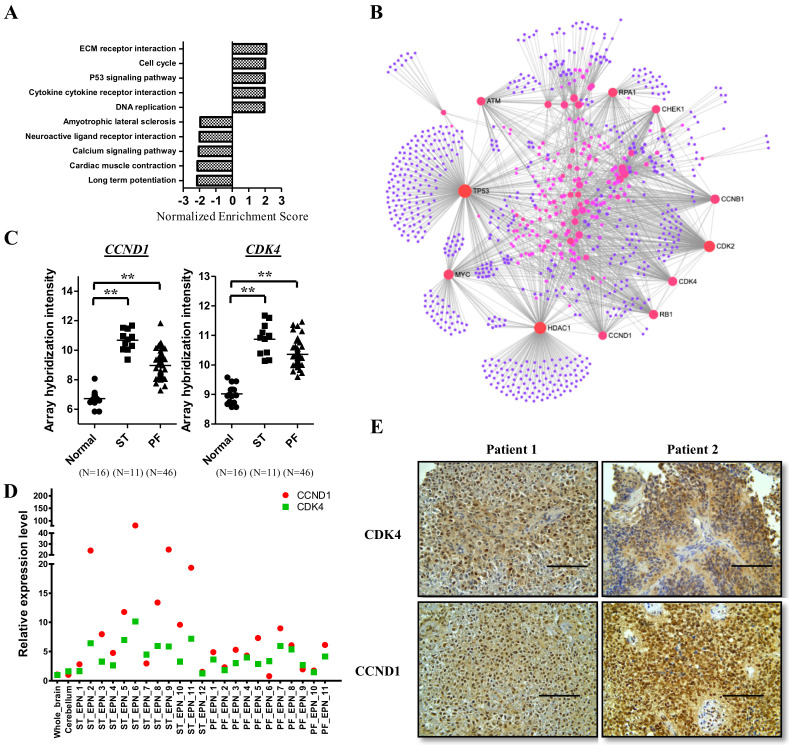
CDK4 and CCND1 are overexpressed in ependymomas. (**A**) Whole genes were subjected to gene set enrichment analysis (GSEA). The bar graph shows the top five upregulated and downregulated KEGG pathways in ependymomas. (**B**) The genes upregulated in the cell cycle and DNA replication were analyzed by NetworkAnalyst (https://www.networkanalyst.ca/). (**C**–**E**) Validation of CDK4 and CCND1 expression by qPCR (**D**) and IHC staining (**E**). Scale bar: 100 μm. The array hybridization signals are also shown (**C**). ** *p* < 0.01. ST: supratentorial, PF: posterior fossa, also known as infratentorial.

Because the recurrence of ependymomas reduces OS and quality of life, and because ependymomas are resistant to currently used chemotherapies, it is crucial to explore the significant pathways underlying ependymoma pathogenesis to identify possible target genes for the development of new therapies to treat this malignant tumor. We combined our microarray datasets [2] with public datasets to analyze the profiles of genes differentially expressed in ependymomas, and loaded all genes into the gene set enrichment analysis (GSEA) to analyze major KEGG enrichment pathways in ependymomas. The top five upregulated and downregulated KEGG pathways are shown in Figure 2A. Upregulation pathways in tumors included ECM–receptor interaction, the cell cycle, the p53 signaling pathway, DNA replication, cytokines, and cytokine–receptor interaction. Downregulation pathways in tumors included long-term potentiation, cardiac muscle contraction, the calcium signaling pathway, neuroactive ligand–receptor interaction, and amyotrophic lateral sclerosis. To explore therapeutic targets, we focused on the upregulated pathways. The data revealed that cell cycle regulation and DNA replication are the most crucial pathways in the control of tumor growth. Therefore, we selected upregulated genes from these two pathways and analyzed the gene network to find the major hotspots. Using gene network analysis, 11 hotspot genes were determined, namely ATM, RPA1, CHEK1, CCNB1, CDK2, CDK4, RB1, CCND1, HDAC1, MYC, and TP53 (Figure 2B). CDK4, CCND1, and RB1 were found to be upstream regulators in the cell cycle process; therefore, CDK4 and CCND1 seem to be the main targets in ependymomas. To verify whether CDK4 and CCND1 are overexpressed in ependymomas, we first checked the expression levels of CCND1 and CDK4 in the microarray dataset. The array hybridization intensity showed that CDK4 and CCND1 are significantly upregulated in supratentorial and posterior fossa ependymomas (Figure 2C). Next, we examined the expression levels of CDK4 and CCND1 using qPCR and IHC staining of clinical samples from the Taipei Veterans General Hospital (VGH). Compared with whole brain and cerebellum tissues, all patients with supratentorial ependymomas (12 patients) and posterior fossa ependymomas (11 patients) exhibited at least one target that was upregulated according to their mRNA levels (Figure 2D). IHC staining also showed overexpression of CDK4 and CCND1 in our clinical samples (Figure 2E).

### 2.2. The CDK4/6 Inhibitor, Abemaciclib, Reduced Cell Proliferation and Caused Cell Death in Ependymomas

**Figure 3:**
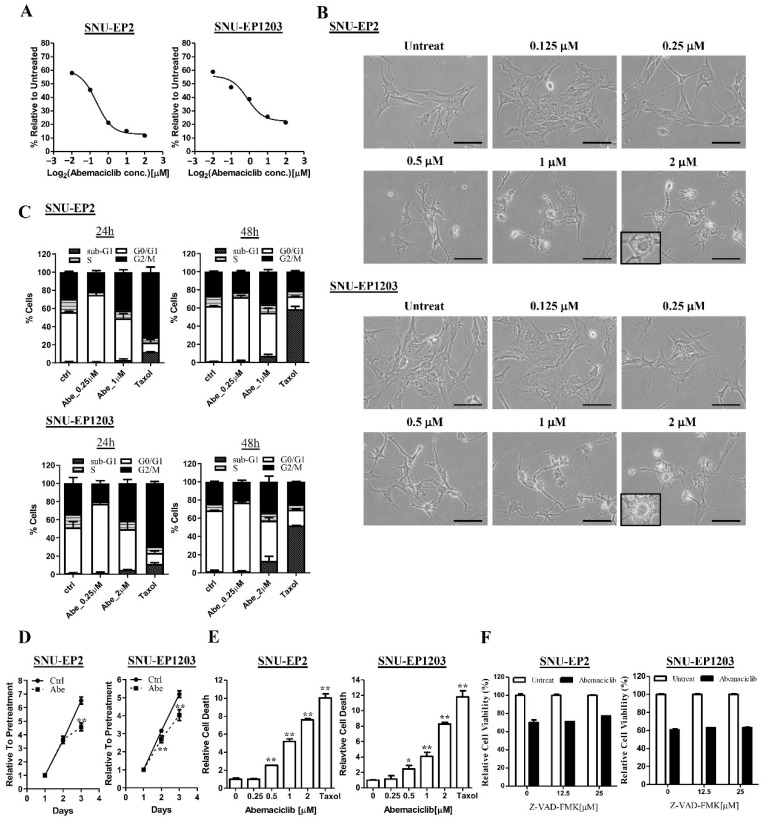
Abemaciclib treatment regulates cell proliferation and cell death in ependymoma cells. (**A**) SNU-EP2 and SNU-EP1203 cells were treated with abemaciclib for 72 h and the IC_50_ value was evaluated by using MTT assay. The IC_50_ value was 0.82 µM in SNU-EP2 and 0.89 µM in SNU-EP1203. Data are presented as the mean ± SD of triplicate wells and are representative of two independent experiments. (**B**) SNU-EP2 and SNU-EP1203 cells were treated with different concentrations of abemaciclib for 24 h and their morphology was examined by phase-contrast microscopy. Scale bar: 100 µm. (**C**) SNU-EP2 and SNU-EP1203 cells were exposed to the indicated concentrations of abemaciclib for 24 and 48 h and then subjected to cell cycle analysis. Data are presented as the mean ± SD (*n* = 2). Abe: abemaciclib. (**D**) Abemaciclib treatment (0.25 µM) reduced the cell proliferation rate in SNU-EP2 and SNU-EP1203 cells. ** *p* < 0.01. (**E**) SNU-EP2 and SNU-EP1203 cells were treated with different concentrations of abemaciclib and Taxol for 72 h and the cells were stained with trypan blue and dead cells were counted using the LUNA II cell counter. The number of dead cells was normalized to the untreated group. Data are presented as the mean ± SD of duplicate samples and are representative of three independent experiments. * *p* < 0.05, ** *p* < 0.01. (**F**) SNU-EP2 and SNU-EP1203 cells were treated with abemaciclib (1 µM) combined with the indicated concentration of Z-VAD-FMK for 72 h, and cell viability was assessed by using MTT assay. Data are presented as the mean ± SD of duplicate samples and are representative of three independent experiments.

Since February 2016, the FDA has approved three CDK4/6 inhibitors—palbociclib, ribociclib, and abemaciclib—to treat women with HR-positive, HER2-negative advanced, and metastatic breast cancers with disease progression. In support of the strategy of targeting the CDK4/6–cyclin D1 complex for more effective treatment of ependymomas, a study demonstrated that abemaciclib, compared to other CDK4/6 inhibitors, has a higher efficiency in passively crossing the BBB [20]. Therefore, we evaluated the treatment efficiency of abemaciclib on SNU-EP2 and SNU-EP1203 cells. We treated SNU-EP2 and SNU-EP1203 cells with different concentrations of abemaciclib and determined their IC_50_ values using an MTT assay. The IC_50_ values of abemaciclib in SNU-EP2 and SNU-EP1203 cells were 0.82 and 0.89 µM, respectively (Figure 3A). Notably, we observed a significant morphological change after 24 h of treatment. Abemaciclib treatment caused cytoplasmic retraction in a concentration-dependent manner (Figure S1A,B), and we also observed cytoplasmic vacuoles following treatment with a high concentration of abemaciclib (2 µM) (Figure 3B). Next, we examined the effect of abemaciclib on cell cycle progression by using propidium iodide (PI) staining and flow cytometry. The data reveal that treatment with a low concentration of abemaciclib increased the accumulation of cells at the G1 phase at 24 and 48 h (Figure 3C and Figure S2). However, a fraction of ependymoma cells treated with a high concentration of abemaciclib were arrested at the G2 rather than G1 phase at 24 and 48 h (Figure 3C and Figure S2). The MTT assay also demonstrated that treatment with a low concentration of abemaciclib delayed the cell proliferation rate in the abemaciclib-treated cells compared with the control cells (Figure 3D). Taxol treatment caused G2 arrest at 24 h and induced sub-G1 apoptosis at 48 h. To determine whether G2 arrest induced by treatment with a high concentration of abemaciclib also induced cell death, we examined the proportion of sub-G1 phase cells and counted dead cells by trypan blue staining. The cell cycle analysis revealed that few sub-G1 phase cells were found following treatment with a high concentration of abemaciclib (Figure 3C). However, abemaciclib treatment increased the population of trypan-blue-stained cells in a concentration-dependent manner (Figure 3E). To investigate whether abemaciclib-induced cell death was caspase-dependent, we analyzed the effect of the pan-caspase inhibitor Z-VAD-fmk combined with 1 μM abemaciclib. As shown in Figure 3F, Z-VAD-fmk did not reduce the cytotoxicity induced by abemaciclib in ependymoma cells. These results indicate that treatment with abemaciclib not only restricted cell proliferation but also caused cell death in ependymoma cells.

**Figure 4:**
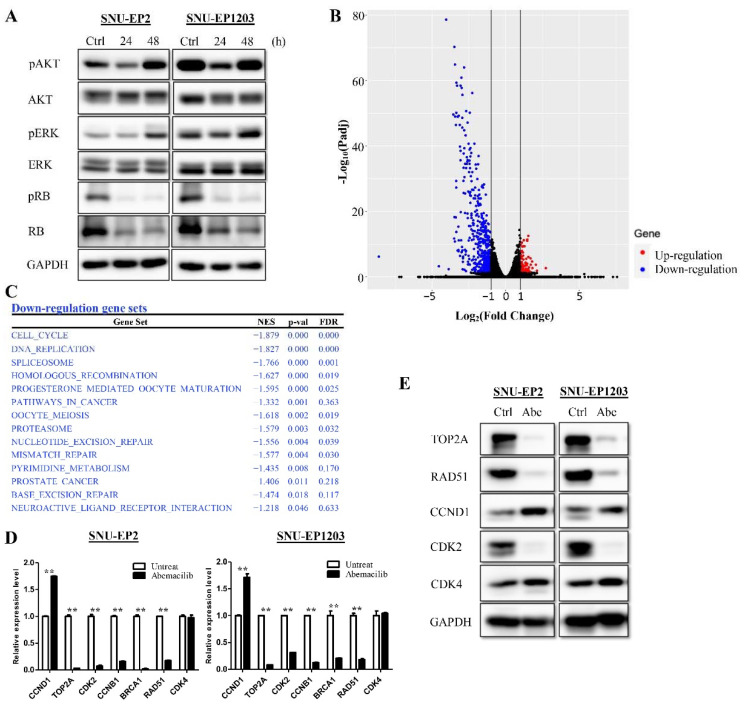
Abemaciclib treatment regulates expression of cell cycle and DNA repair genes. (**A**) SNU-EP2 and SNU-EP1203 cells were treated with abemaciclib (0.25 µM) for the indicated times and the levels of phosphorylated RB, AKT, and ERK were detected via Western blot analysis. (**B**) SNU-EP2 cells were treated with abemaciclib for 48 h and mRNA-seq was performed. A volcano plot showed significantly upregulated (red) and downregulated (blue) genes. (**C**) Significantly downregulated pathways analyzed by GSEA are listed. (**D**,**E**) Expression of cell cycle and DNA repair genes in SNU-EP2 and SNU-EP1203 cells was validated by qPCR (**D**) and Western blot analysis (**E**). qPCR results are presented as the mean ± SD for duplicate samples. ** *p* < 0.01.

Treatment with a low concentration of abemaciclib was sufficient to delay proliferation in ependymoma cells. However, SNU-EP2 and SNU-EP1203 cells still proliferated slowly. To determine whether cell growth-related pathways were also affected by abemaciclib, we examined the phosphorylation status of RB, AKT, and ERK. SNU-EP2 and SNU-EP1203 cells were treated with 0.25 µM abemaciclib for 24 and 48 h. The levels of p-AKT(Ser473), p-ERK(Thr202/Tyr204), and p-RB(Ser807/811) were detected by Western blot analysis. Treatment of cells with abemaciclib inhibited the phosphorylation of AKT and ERK at 24 h. Inhibition of RB phosphorylation persisted for 48 h after treatment. By contrast, phosphorylation of AKT and ERK had recovered at 48 h (Figure 4A). Furthermore, we found that abemaciclib treatment reduced total RB expression. We further investigated which molecules were affected in terms of expression after abemaciclib treatment. mRNA sequencing of SNU-EP2 cells treated with abemaciclib, or vehicle control for 48 h was performed, and alterations in global expression were analyzed. A total of 83 genes had increased expression and 455 genes had decreased expression after abemaciclib treatment (Padj < 0.05, fold change ≥ 2) (Figure 4B). GSEA analysis revealed that the genes involved in the cell cycle, DNA replication, and several DNA repair mechanisms were particularly suppressed by abemaciclib treatment (Figure 4C). We verified the expression of multiple genes using qPCR and Western blot analyses of SNU-EP2 and SNU-EP1203 cells and demonstrated a decrease in the mRNA levels of *CCNB1*, *TOP2A*, *CDK2*, *BRCA1*, and *RAD51* after abemaciclib treatment (Figure 4D). The protein levels of TOP2A, RAD51, and CDK2 also decreased (Figure 4E). By contrast, the mRNA levels of CCND1 increased while those of CDK4 were unchanged after abemaciclib treatment (Figure 4D). The protein levels of CCND1 and CDK4 increased after abemaciclib treatment (Figure 4E). These data suggest that abemaciclib inhibits cell proliferation by inhibiting RB, AKT, and ERK phosphorylation and through downregulating the expression of genes related to the cell cycle, DNA replication, and DNA repair.

**Figure 5:**
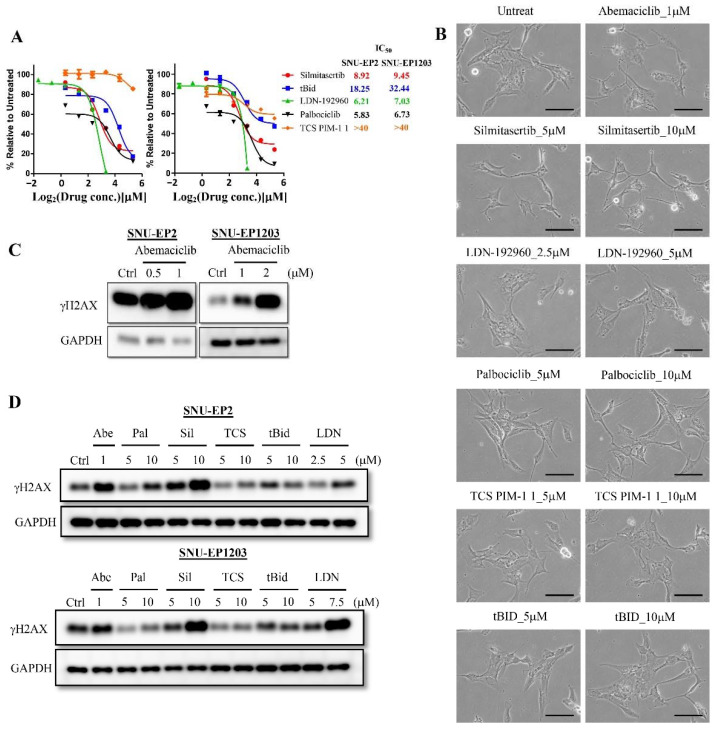
Abemaciclib treatment mediates morphological changes and γH2AX expression through CK2 and DYRK2 in ependymoma cells. (**A**) SNU-EP2 and SNU-EP1203 were treated with specific inhibitors for 72 h and IC_50_ values were evaluated by MTT assay. The IC_50_ values are shown on the right side of the graph. (**B**) SNU-EP2 was exposed to the indicated concentrations of specific inhibitors for 24 h and morphology was examined by phase-contrast microscopy. Scale bar: 100 µm. (**C**) SNU-EP2 and SNU-EP1203 were treated with different concentrations of abemaciclib for 72 h, and the levels of γH2AX were detected via Western blot analysis. (**D**) SNU-EP2 and SNU-EP1203 were exposed to the indicated concentrations of specific inhibitors for 24 h, and the levels of γH2AX were detected via Western blot analysis. Pal: palbociclib, Sil: silmitasertib, TCS: TCS PIM-1 1, LDN: LDN-192960.

A previous study showed that abemaciclib has multiple targets, including CDK4, CDK6, DYRK2, Pim1, HIPK2, and CK2 [21]. To investigate whether abemaciclib causes cytoplasmic retraction through targeting of CDK4/6 or other targets, we treated SNU-EP2 and SNU-EP1203 cells with specific inhibitors of CDK4/6 (palbociclib), DYRK2 (LDN-192960), PIM1 (TCS Pim-1 1), HIPK2 (tBid), and CK2 (silmitasertib) and determined their IC_50_ values. The average IC_50_ values of palbociclib, LDN-192960, tBid, and silmitasertib were found to be 6.28 ± 0.64, 6.62 ± 0.58, 25.35 ± 10.03, and 9.19 ± 0.37 μM in SNU-EP2 and SNU-EP1203 cells, respectively (Figure 5A). However, the PIM1 inhibitor, TCS Pim-1 1, did not delay cell proliferation nor cause cell death (IC_50_ > 40 µM). We further treated different concentrations of five inhibitors for 24 h and observed that silmitasertib treatment presented a similar cytoplasmic retraction effect to abemaciclib treatment (Figure 5B and Figure S3). A previous study showed that abemaciclib treatment alone or in combination with radiation causes γH2AX expression in non-small lung cancer cell lines [22]. To check whether abemaciclib treatment also induces γH2AX expression in ependymomas, we treated SNU-EP2 and SNU-EP1203 cells with different concentrations of abemaciclib for 72 h and determined γH2AX expression. We found that abemaciclib treatment was associated with increased γH2AX levels in a concentration-dependent manner (Figure 5C). To determine whether abemaciclib induced γH2AX expression through targeting CDK4/6 or other targets, we treated different concentrations of five inhibitors for 24 h and determined γH2AX expression. We found that increasing concentrations of LDN-192960 and silmitasertib were associated with greater γH2AX levels (Figure 5D). These data suggest that abemaciclib-induced morphology changes and γH2AX expression may occur through targeting CK2 and DYRK2, not CDK4, CDK6, HIPK2, or PIM1, in ependymomas.

### 2.3. Abemaciclib Causes Significant Regression of Subcutaneous Patient-Derived Ependymoma Tumors

Surgical residual tumor samples from a 1-year-old ependymoma patient were implanted into a subcutaneous pocket made on each side of the lower backs of NOD-SCID mice. Tumors were excised after reaching a size of 1–2 cm^3^. Parts of the tumor samples were subsequently implanted into mice, and the remaining portions of the tumors were used for validation by employing IHC staining and mRNA sequencing. Sections of primary ependymomas were typical of ependymomas, featuring a fibrillary background, monomorphic cells, round to ovoid nuclei with numerous perivascular pseudorosettes (nuclear-free zones), and scant true ependymal rosettes. Features of anaplastic ependymoma, such as increased mitotic activity and hypercellularity, were noted in more than 70% of the area. Sections of all generations of PDXs showed the same morphology as that of anaplastic ependymomas. Sections of first-generation PDX (PDX_P1) showed especially brisk mitotic activity (>5–10 mitoses per 10 HPF). The fibrillary background and tumor cell process toward perivascular pseudorosettes can be highlighted by the biomarker of the glial lineage tumor, GFAP (Figure S4A). mRNA sequencing was performed to check whether all generations exhibited expression patterns similar to the primary tumor in addition to identifying the expression patterns of the primary tumor and all generations of the tumor. Sequencing data of pediatric glioblastomas were included in comparative transcriptome analysis. As expected, principal component analysis (PCA) and unsupervised clustering analysis showed that xenografts of all generations were more similar to those of primary ependymomas than other glioblastomas (Figure S4B,C). To determine whether the expression patterns of xenografts were sufficient to represent a primary tumor, we calculated the correlation coefficients, and the results showed that the expression patterns of all xenografts were highly similar to those of primary tumors (*r* = 0.86–0.87) (Figure S4D). Furthermore, the second and third generations exhibited higher correlations to the primary transplants (*r* = 0.97 and *r* = 0.95, respectively).

**Figure 6:**
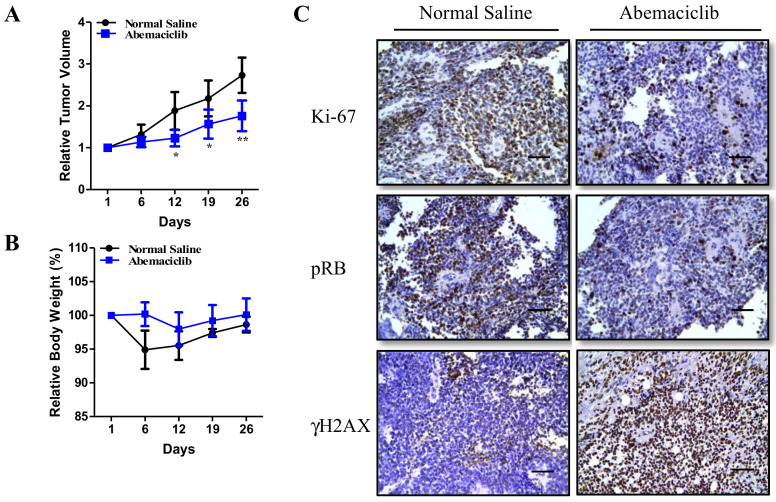
Abemaciclib treatment inhibits patient-derived ependymoma xenografts growth in vivo. (**A**,**B**) The third-generation xenografts were injected subcutaneously into the flank regions of nude mice, and abemaciclib was administered orally (50 mg/kg/day) five times per week. Tumor volume (**A**) and body weight (**B**) were recorded every 6 or 7 days. The figure shows the relative tumor volume and body weight compared with those at the first abemaciclib feeding. * *p* < 0.05, ** *p* < 0.01. (**C**) IHC staining for detection of Ki-67 and γH2AX expression and RB phosphorylation after abemaciclib treatment. Scale bar: 50 µm.

To evaluate whether abemaciclib can inhibit tumor growth in vivo, a fourth generation of mice was established and treated with a vehicle or once daily with oral abemaciclib (50 mg/kg/day, 5 days a week). As shown in Figure 6A, abemaciclib significantly inhibited tumor growth. Furthermore, we compared mouse weights and did not observe any significant toxicity associated with abemaciclib treatment (Figure 6B). To elucidate the molecular mechanisms underlying the tumor-suppressive effect of abemaciclib, the residual xenografted tumors were analyzed using IHC staining. Lower levels of RB phosphorylation and Ki-67 expression and high levels of γH2AX expression were observed after abemaciclib treatment (Figure 6C). In summary, we demonstrated that abemaciclib significantly suppresses cell proliferation and tumor growth, both in vitro and in vivo.

## 3. Discussion

In a previous study, we analyzed a large clinical cohort of ependymomas in pediatric patients treated at Taipei VGH and observed that pediatric ependymomas were associated with worse outcomes despite aggressive neuro-oncological treatment. The long-term 10-year OS and progression-free survival (PFS) rates were approximately 60% and 30%, respectively, for ependymomas [2]. The data reflect the limitations of present therapeutic applications and the high recurrence rates of challenging tumors in children. The benefit of chemotherapy treatment for ependymomas has not yet been demonstrated. Therefore, the development and testing of targeted therapies based on molecular insights is urgently required and crucial in accessing potential new treatment agents for young patients. Previous studies have shown that cyclin D1 overexpression is associated with a poor prognosis and recurrence in supratentorial ependymomas [23,24]. In our earlier studies, we also identified incremental cyclin D1 expression in primary and recurring ependymomas and demonstrated that cyclin D1 regulates resistance to radiotherapy in ependymomas [2]. In this study, we collected mRNA microarray data from supratentorial, posterior fossa group A, and posterior fossa group B ependymomas and performed differential gene expression and pathway analyses. We found that most cell-cycle-related genes were overexpressed in supratentorial and posterior fossa ependymomas, particularly cyclin D1 and CDK4, which can serve as actionable therapeutic targets. 

The FDA recently approved three CDK4/6 inhibitors—palbociclib, ribociclib, and abemaciclib—for the treatment of HR-positive, HER2-negative advanced, or metastatic breast cancers. These three inhibitors mainly bind to the ATP-binding pocket of CDK4 and CDK6, and all have greater affinity for CDK4 and CDK6 than other CDKs [14]. Preclinical studies have demonstrated that CDK4/6 inhibitors, particularly palbociclib and abemaciclib, have similar levels of antitumor activity. Palbociclib was shown to effectively arrest cell cycle progression and tumor growth in several cancers, including hepatocellular carcinoma [25,26], glioblastoma [15,16,27], synovial sarcoma [28], medulloblastoma [19,29], AT/RTs [18], and gastric cancers [30]. Abemaciclib was also found to effectively induce cell cycle arrest and tumor growth inhibition in several cancers, including non–small cell lung cancer [22], glioblastoma [17], Ewing’s sarcoma [31], multiple myeloma [32], and aggressive germinal center-derived B-cell lymphomas [33]. In this study, we demonstrated that abemaciclib treatment resulted in decreased expression of genes related to the cell cycle and DNA repair through inhibiting RB phosphorylation and total RB protein levels. That abemaciclib not only inhibits RB phosphorylation but also total RB levels was also observed in previous studies [34,35]. Phosphorylation of the RB protein causes dissociation of the E2F protein, which then translocates to the nucleus to regulate target gene expression [36]. E2F-regulated genes are involved in many cellular processes, including the cell cycle, DNA replication, DNA repair, DNA checkpoint, apoptosis and differentiation [37]. In our qPCR and Western blot analyses, CDK2 and CCNB1 expression were downregulated, causing cells to accumulate in the G1 phase. A high dose of abemaciclib caused G2 arrest in ependymoma cells. This phenomenon has also been observed in breast cancer cell lines. A possible mechanism for this is through inhibition of CDK1 and CDK2 expression, whose activities are required for the S and mitosis phases [38]. In addition to inhibition of cell proliferation, abemaciclib treatment also caused cell death in ependymoma cells. In our cell cycle analysis, abemaciclib treatment was associated with a reduction in sub-G1 phase cells. Furthermore, treatment with pan-caspase inhibitor did not prevent abemaciclib-induced cell death. These data suggest that abemaciclib causes caspase-independent cell death in ependymoma cells. Abemaciclib-induced caspase-independent cell death has also been found in multiple myeloma cells [32], and another study suggested that abemaciclib induces lysosomal swelling and dysfunction, resulting in atypical cell death [39]. Cytoplasmic vacuoles were also observed in SNU-EP2 and SNU-EP1203 cells after 24 h of abemaciclib treatment. Therefore, whether SNU-EP2 and SNU-EP1203 cells undergo atypical cell death after abemaciclib treatment will need to be clarified in the future.

In addition to CDK4 and CDK6, abemaciclib inhibits CDK9 and non-CDK targets, such as DYRK2, PIM1, HIPK2, and CK2 [21]. In this study, we found that abemaciclib could induce γH2AX expression in SNU-EP2 and SNU-EP1203 cells. Through using specific inhibitors for CDK4, CDK6, DYRK2, PIM1, HIPK2, and CK2, our data demonstrate that the inhibition of DYRK2 and CK2 induced greater γH2AX expression and that the inhibition of CK2 also caused cytoplasmic retraction. CK2 is a highly conserved Ser/Thr kinase composed of two catalytic α subunits and two regulatory β subunits. CK2 is involved in several cellular processes, including regulation of the cell cycle, survival, DNA sensing and repair, and maintenance of the cytoskeleton [40,41]. A previous study showed that knockdown of CK2 expression combined with neocarzinostatin treatment induced higher γH2AX expression [40]. Furthermore, another study found that inhibition of CK2 caused cytoplasmic retraction and cell rounding in cells [41,42,43]. In this study, we showed that abemaciclib treatment is associated with γH2AX expression and cytoplasmic retraction, and silmitasertib treatment also resulted in similar phenomena; therefore, we think that one possible mechanism through which abemaciclib causes cell death is through targeting of CK2. Another possible mechanism is the targeting of DYRK2. DYRK2 is also a Ser/Thr kinase and is involved in numerous cellular processes, including cell cycle progression, cell migration, and apoptosis [44]. DYRK2 is also involved in double-strand break (DSB) repair. In response to DNA damage, DYRK2 is stabilized and translocates to the nucleus following phosphorylation by ATM. Phosphorylated DYRK2 phosphorylates p53 at residue 46, suggesting that it is involved in p53-dependent apoptosis [45]. Knockdown of DYRK2 impairs HR efficacy and decreases the monoubiquitination of γH2AX [44,45]. These data also suggest that abemaciclib possibly causes cell death through targeting DYRK2. However, the detailed mechanism still requires future investigation.

In this study, abemaciclib delayed the proliferation of SNU-EP2 and SNU-EP1203 cells and patient-derived ependymoma xenograft growth. However, we found that SNU-EP2 and SNU-EP1203 cells and patient-derived ependymoma xenografts continued to proliferate after abemaciclib treatment, although the proliferation rate was lower than that of control samples. These results indicate that a lower concentration of abemaciclib might not cause sufficient cell death in ependymomas. Higher concentrations of drugs often cause toxicity and severe side effects in the clinical setting. Therefore, if abemaciclib treatment alone is not enough to decrease tumor volume, a combination of CDK4/6 inhibitors with chemotherapy or radiotherapy is essential. In our Western blot data, we observed recovery of p-AKT and p-ERK after 48 h of abemaciclib treatment. Therefore, a combination of drugs targeting the AKT or ERK pathways along with abemaciclib may be successful in preventing continuous cell proliferation. Previous preclinical studies have shown that a combination of CDK4/6 inhibitors, palbociclib, or abemaciclib with everolimus or altiratinib resulted in longer OS than treatment with CDK4/6 inhibitors alone in glioblastoma mouse models [15,17]. In addition, combining CDK4/6 inhibitors with chemotherapy and radiotherapy was more beneficial to OS than treatment with CDK4/6 or radiation alone in mouse models of AT/RTs, glioblastoma, and non-small cell lung cancer [16,18,22]. According to our RNA sequencing data, DNA repair genes, such as *RAD51* and *BRCA2*, were downregulated after abemaciclib treatment. Our previous study demonstrated that knockdown of cyclin D1 expression resulted in lower *RAD51* and *BRCA2* expression and suppressed the ability of cells to undergo homologous recombination [2]. These results indicate that the combination of radiotherapy with abemaciclib is possibly beneficial for the treatment of ependymomas. To determine whether combination therapy is beneficial for ependymoma treatment, we need to establish more patient-derived ependymoma xenografts for further study.

## 4. Materials and Methods

### 4.1. Cell Culture

Two types of human primary ependymoma cell lines, SNU-EP2 and SNU-EP1203, were established and obtained from Dr. Seung-Ki Kim and Dr. Ji-Hoon Phi’s laboratory (Seoul National University Children’s Hospital, Seoul, Republic of Korea) [46]. SF188 and KNS42 cells were maintained in Dulbecco’s modified Eagle medium-F12 (DMEM/F12) and ependymoma cells were kept in DMEM (Gibco/Life Technologies, Carlsbad, CA, USA) supplemented with 10% fetal bovine serum (Gibco/ Life Technologies). These cells were incubated at 37 °C in a humidified atmosphere of 5% CO_2_.

### 4.2. Biological Samples

The parents or legal guardians of the patients whose cells were used in this study provided informed consent, and all procedures were approved by the Institutional Review Board of Taipei Veterans General Hospital (VGH-TPE) (VGHIRB No.: 2013-07-016A (approval period: 2013/07/12-2014/07/11), 2013-01-020B (approval period: 2013/02/22-2014/02/21), 2014-05-006C (approval period: 2014/06/06-2015/06/05), 2015-12-008A (approval period: 2016/01/12-2017/01/11), 20l6-05-007C (approval period: 2016/06/14-2017/06/13) and 2016-07-001C (approval period: 2016/07/20-2017/07/19)). Fresh tumor tissues were collected during surgery in patients with ependymomas and frozen until use. Surgical pathology data were retrieved from the Department of Pathology and Laboratory Medicine at Taipei VGH.

### 4.3. Microarray and RNA-Seq Analysis

Public microarray datasets were obtained from our previous studies [2] and the Gene Expression Omnibus datasets GSE35493, GSE66354, and GSE68015 were used. Microarray analysis was performed as previously described [47]. For mRNA, high-throughput sequencing was used, and total RNA was extracted from cells and tissues. RNA quality and quantity were assessed using a bioanalyzer and Qubit, and the RNA was then ligated to an adaptor for further amplification (Illumina^®^ TruSeq™ stranded mRNA, San Diego, CA, USA). All library preparation was performed in the translational core facility of Taipei Medical University. After sequencing was completed, the reads files (fastq) were mapped to the Hg19 reference using STAR, and gene expression was determined using RSEM. Differential genes were identified using the R package, DESeq2, and gene ontology software, IPA, GSEA, and DAVID GO (https://david.ncifcrf.gov/) were used to decipher gene function.

### 4.4. RNA and Reverse Transcription-Quantitative PCR

Total RNA from tumor tissues and cultured cells was isolated using TRIzol reagent (Invitrogen/Life Technologies, Carlsbad, CA, USA). Total RNA was reverse-transcribed into complementary DNA through random hexamer priming using a HiScript II Q RT SuperMix (Vazyme, Nanjing, China). Quantitative PCR was performed in duplicate with gene-specific primers using a Maxima™ SYBR FAST qPCR kit (Thermo Scientific, Grand Island, NY, USA).

### 4.5. Immunoblotting and Immunohistochemistry

Immunoblotting was performed using anti-phospho-RB (Cell Signaling, Danvers, MA, USA), anti-RB (GeneTex, Irvine, CA, USA), anti-phospho-Akt(Ser473) (Cell Signaling), anti-Akt (Cell Signaling), anti-phospho-Erk1/2(Thr202/Tyr204) (Cell Signaling), anti-Erk1/2 (Cell Signaling), anti-TOP2A (Cell Signaling), anti-CCND1 (Cell Signaling), anti-γH2AX (Cell Signaling), anti-CDK2 (GeneTex), anti-RAD51 (GeneTex), anti-CDK4 (Cell Signaling), and anti-GAPDH (GeneTex) antibodies followed by horseradish peroxidase-conjugated secondary antibodies and visualization using an enhanced chemiluminescence detection system. Immunohistochemical (IHC) sample preparation and staining with anti-GFAP (Genetex), anti-phospho-RB (Cell Signaling), anti-Ki-67 (Cell Signaling), and anti-γH2AX (Cell Signaling) antibodies were carried out as previously described [48]. Uncropped Western blot images are provided in Figure S5.

### 4.6. MTT Assay

To evaluate cell viability, cells were seeded at a concentration of 3 × 10^3^/well and incubated at 37 °C. After incubation for 24 h, the cells were treated with different concentrations of abemaciclib. After incubation for 24, 48, and 72 h, the cells were treated with 1% thiazolyl blue tetrazolium for 1 h at 37 °C followed by addition of 0.1% sodium dodecyl sulfate in 2-propanol and thorough mixing. The results were obtained by measuring absorbance at 570 and 650 nm using a multiwell scanning spectrophotometer.

### 4.7. Cell Cycle Analysis

Cell cycle analysis was performed as previously described [49]. Cells were seeded in 6-well plates and exposed to the indicated compounds for 24 to 48 h. The cells were collected by trypsinization, washed by PBS, and fixed with ethanol (70%) at −20 °C overnight. The cells were pelleted by centrifugation and incubated in 0.1 mL of phosphate–citric acid buffer (0.2 M NaHPO_4_ and 0.1 M citric acid, pH 7.8) for 15 min at room temperature and then resuspended in propidium iodide staining buffer containing Triton X-100 (0.1%, *v*/*v*), RNase A (100 μg/mL), and propidium iodide (80 μg/mL) and incubated for 30 min in the dark. Cell cycle distribution was analyzed by using flow cytometry with CellQuest software (Becton Dickinson, Mountain View, CA, USA).

### 4.8. PDX Mouse Establishment and Treatment

All animal experiments were approved by the institutional animal care and use committee (IACUC) of VGH-TPE (IACUC No.: IACUC 2017-077(approval period: 2018/1/1-12/31), IACUC 2018-073 (approval period: 2019/1/1-12/31)). In general, pediatric brain tumor tissues were diced into 2 × 2 × 3 mm^3^ fragments and implanted into 6–8 week old male NOD-SCID mice (F1) by making a small incision and a subcutaneous pocket on each side of the lower back. The tumor was removed for serial implantation after it had grown to approximately 1–2 cm^3^. The tumor was additionally passaged two times in NOD/SCID mice (F2 and F3), and the implantation was performed under sterile conditions. The tumor samples from the passage (F1–F3) were stored in liquid nitrogen and used for further experiments, such as retransplantation. The tumor tissues (F1–F3) were analyzed through hematoxylin and eosin staining, and the tumor samples, confirmed by a pathologist, were subjected to gene expression profile analysis and compared with the original tumors. The fourth generation of mice was established for abemaciclib treatment experiments. When the tumors grew to approximately 0.8 cm^3^, the mice were stratified into two groups and randomly assigned to be treated with normal saline or abemaciclib (dissolved in normal saline, 50 mg/kg/day, given 5 days a week). The treatment was administered for 1 month. Tumor volumes and mouse weights were recorded every 6 or 7 days. Tumor volume was calculated as tumor volume (mm^3^) = [(length × width^2^)/2].

### 4.9. Statistical Analysis

For the in vitro and in vivo experiments, two-tailed Student’s *t* tests were used to assess the significance of mean differences. Differences were considered significant at *p* < 0.05. All data are reported as the mean ± standard deviation.

## 5. Conclusions

In this study, integrated transcriptome analysis demonstrated that the cell cycle-related genes CDK4 and CCND1 are upregulated in ependymomas. The CDK4/6 inhibitor abemaciclib showed promising therapeutic effects in primary ependymoma cells and in a preclinical ependymoma animal model and, thus, represents a potential therapeutic strategy. Furthermore, the phase I study on the use of abemaciclib and radiation therapy for the treatment of diffuse intrinsic pontine gliomas and recurrent or refractory solid tumors in children is ongoing (NCT02644460, actively enrolling). Additional clinical trials using concurrent chemoradiation therapy or combined multiple chemotherapeutic agents are recommended to determine how to treat challenging tumors in children.

## Supplementary Materials

The following are available online at https://www.mdpi.com/2072-6694/12/12/3597/s1, Figure S1: Abemaciclib treatment mediates changes in cell morphology of SNU-EP2 and SNU-EP1203 ependymoma cells. (A,B) SNU-EP2 and SNU-EP1203 cells were exposed to the indicated concentrations of abemaciclib for 24 h, and morphology was examined in a large field by phase-contrast microscopy. Scale bar: 100 µm. We calculated the area of three fields from each group and then normalized this to the cell number analyzed by using crystal violet staining. The bar chart shows each group normalized to the untreated group. Data are presented as the mean ± SD in three fields and are representative of two independent experiments, Figure S2: Abemaciclib treatment delayed cell cycle progression in SNU-EP2 and SNU-EP1203 ependymoma cells. (A,B) SNU-EP2 and SNU-EP1203 cells were exposed to the indicated concentrations of abemaciclib and Taxol for 24 and 48 h and subjected to cell cycle analysis, Figure S3: Treatment with specific inhibitors mediates cell morphology changes in SNU-EP2 and SNU-EP1203 ependymoma cells. (A) SNU-EP1203 was exposed to the indicated concentrations of specific inhibitors for 24 h, and morphology was examined by phase-contrast microscopy. Scale bar: 100 µm. (B,C) SNU-EP2 and SNU-EP1203 cells were exposed to the indicated concentrations of specific inhibitors for 24 h, and morphology was examined in a large field by phase-contrast microscopy. Scale bar: 100 µm. We calculated the area of three fields from each group and then normalized this to the cell number analyzed by crystal violet staining. The bar chart shows each group normalized to the untreated group. Data are presented as the mean ± SD in three fields and are representative of two independent experiments, Figure S4: Patient-derived ependymoma xenograft validation. (A) IHC analyses confirmed the H&E results and protein levels of GFAP in primary ependymomas and those from three generations. The magnification is 100×. PDX_P1: The first generation. PDX_P2: The second generation. PDX_P3: The third generation. (B,C) The PCA plot (B) and unsupervised clustering analysis (C) based on the total number of genes show the relationships among primary ependymoma, all generations of xenografts, and three glioblastomas (GBMs). (D) The matrix represents the relationships among the primary tumor and all xenografts, and a higher number indicates a closer relationship, Figure S5: Uncropped Western blot images.
