# Supplementary material for: Abemaciclib, A Selective CDK4/6 Inhibitor, Restricts the Growth of Pediatric Ependymomas
Source: Cancers (Basel). 2020 Dec 1;12(12):3597. doi: 10.3390/cancers12123597 (PMC7760843; doi:10.3390/cancers12123597)
Supplement: Supplementary file 1 [file cancers-12-03597-s001.pdf]

Abemaciclib, A Selective CDK4/6 Inhibitor, Restricts the Growth of Pediatric Ependymomas

Muh-Lii Liang, Chun-Han Chen, Yun-Ru Liu, Man-Hsu Huang, Yu-Chen Lin, Tai-Tong Wong, Sey-En Lin, Shing-Shiung Chu, Yi-Huei Ding and Tsung-Han Hsieh

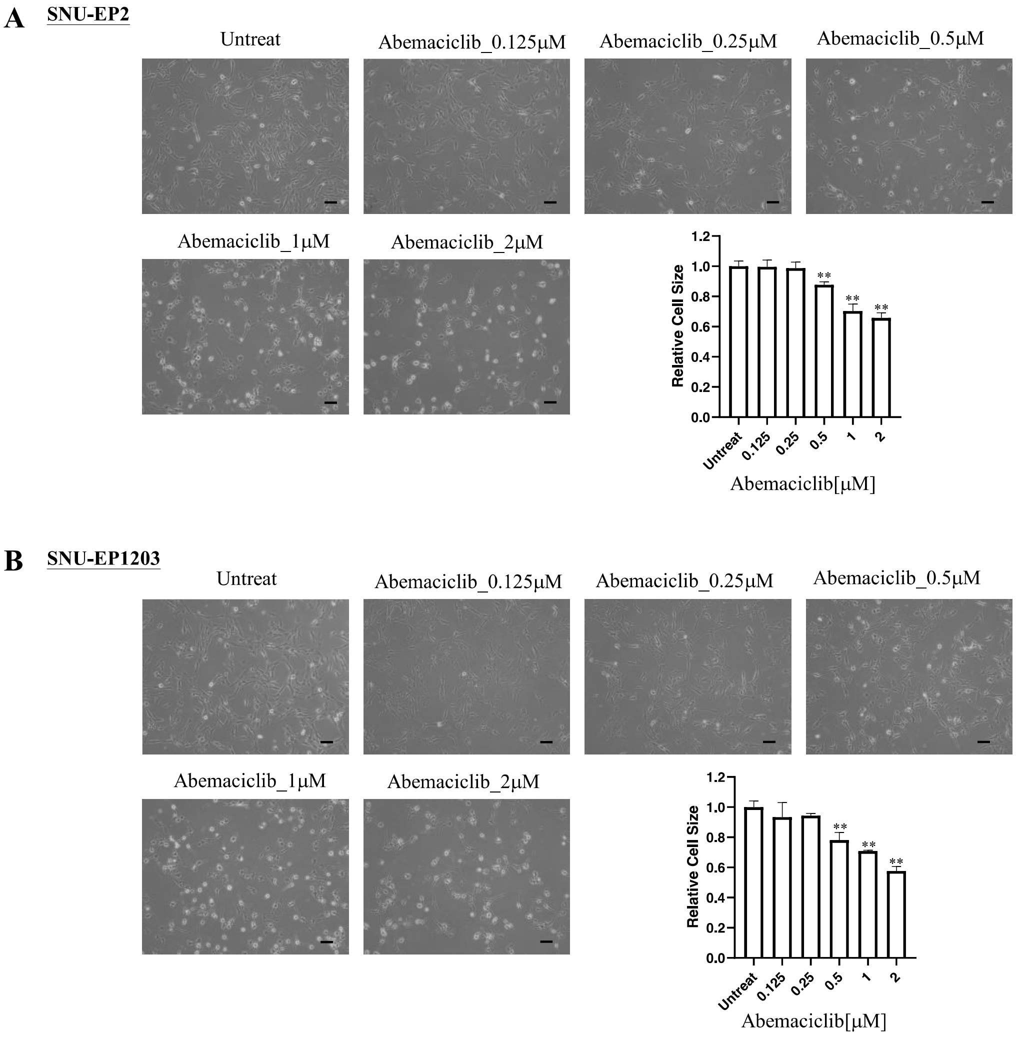

Figure S1. Abemaciclib treatment mediates changes in cell morphology of SNU-EP2 and SNU-EP1203 ependymoma cells. (**A**, **B**) SNU-EP2 and SNU-EP1203 cells were exposed to the indicated concentrations of abemaciclib for 24 h, and morphology was examined in a large field by phase-contrast microscopy. Scale bar: 100 μm. We calculated the area of three fields from each group and then normalized this to the cell number analyzed by using crystal violet staining. The bar chart shows each group normalized to the untreated group. Data are presented as the mean ± SD in three fields and are representative of two independent experiments.

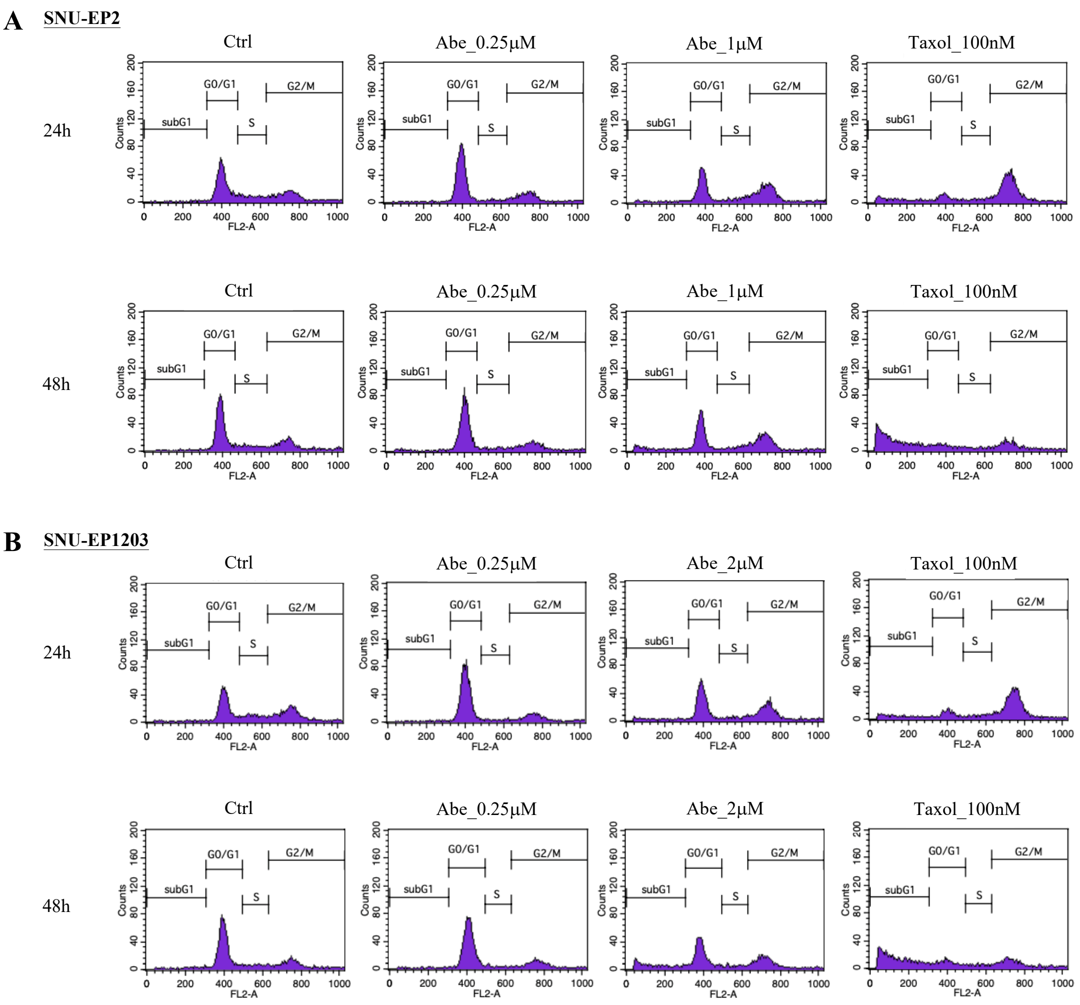

Figure S2. Abemaciclib treatment delayed cell cycle progression in SNU-EP2 and SNU-EP1203 ependymoma cells. (**A**, **B**) SNU-EP2 and SNU-EP1203 cells were exposed to the indicated concentrations of abemaciclib and Taxol for 24 and 48 h and subjected to cell cycle analysis.

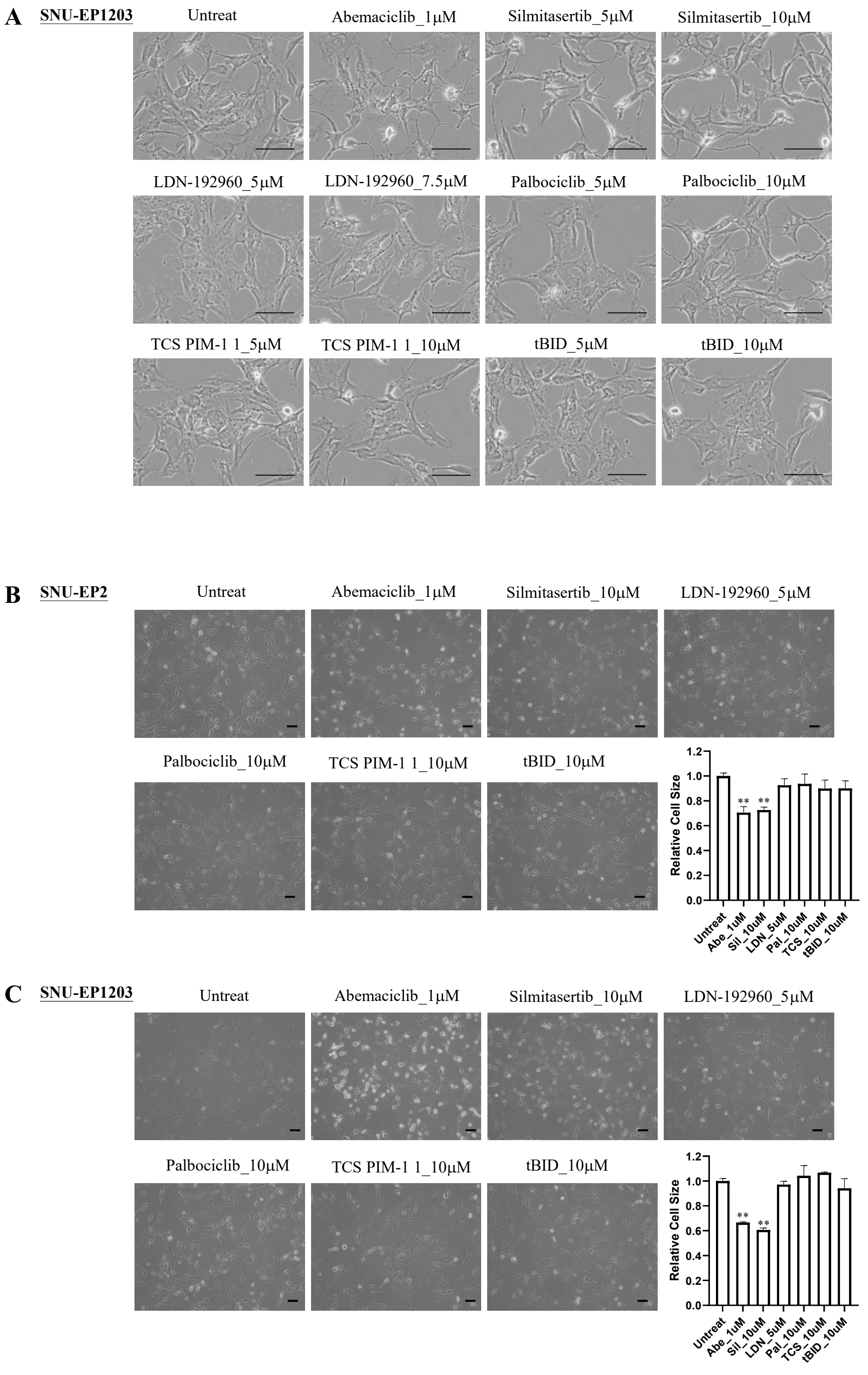

Figure S3. Treatment with specific inhibitors mediates cell morphology changes in SNU-EP2 and SNU-EP1203 ependymoma cells. (**A**) SNU-EP1203 was exposed to the indicated concentrations of specific inhibitors for 24 h, and morphology was examined by phase-contrast microscopy. Scale bar: 100 μm. (**B**, **C**) SNU-EP2 and SNU-EP1203 cells were exposed to the indicated concentrations of specific inhibitors for 24 h, and morphology was examined in a large field by phase-contrast microscopy. Scale bar: 100 μm. We calculated the area of three fields from each group and then normalized this to the cell number analyzed by crystal violet staining. The bar chart shows each group normalized to the untreated group. Data are presented as the mean ± SD in three fields and are representative of two independent experiments.

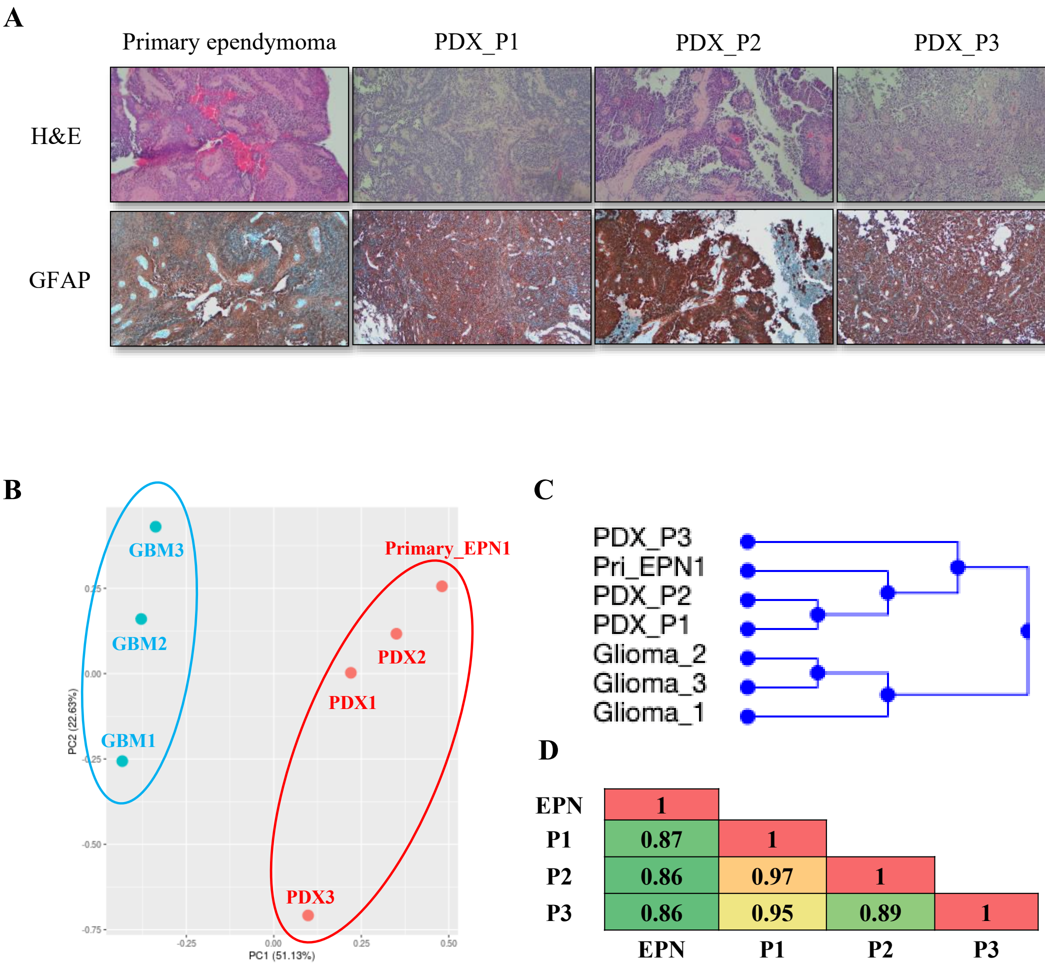

Figure S4. Patient-derived ependymoma xenograft validation. (**A**) IHC analyses confirmed the H&E results and protein levels of GFAP in primary ependymomas and those from three generations. The magnification is 100×. PDX_P1: The first generation. PDX_P2: The second generation. PDX_P3: The third generation. (**B**, **C**) The PCA plot (**B**) and unsupervised clustering analysis (**C**) based on the total number of genes show the relationships among primary ependymoma, all generations of xenografts, and three glioblastomas (GBMs). (**D**) The matrix represents the relationships among the primary tumor and all xenografts, and a higher number indicates a closer relationship.

Figure 4A

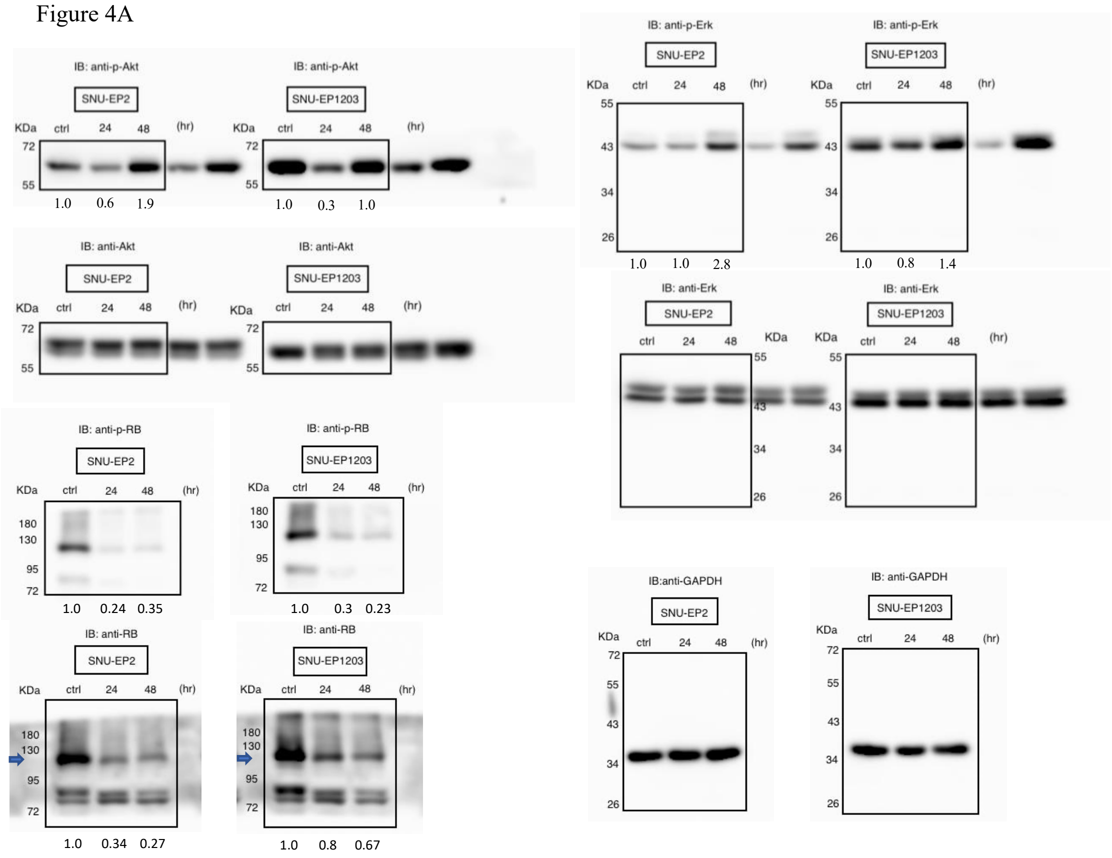

Figure 4E

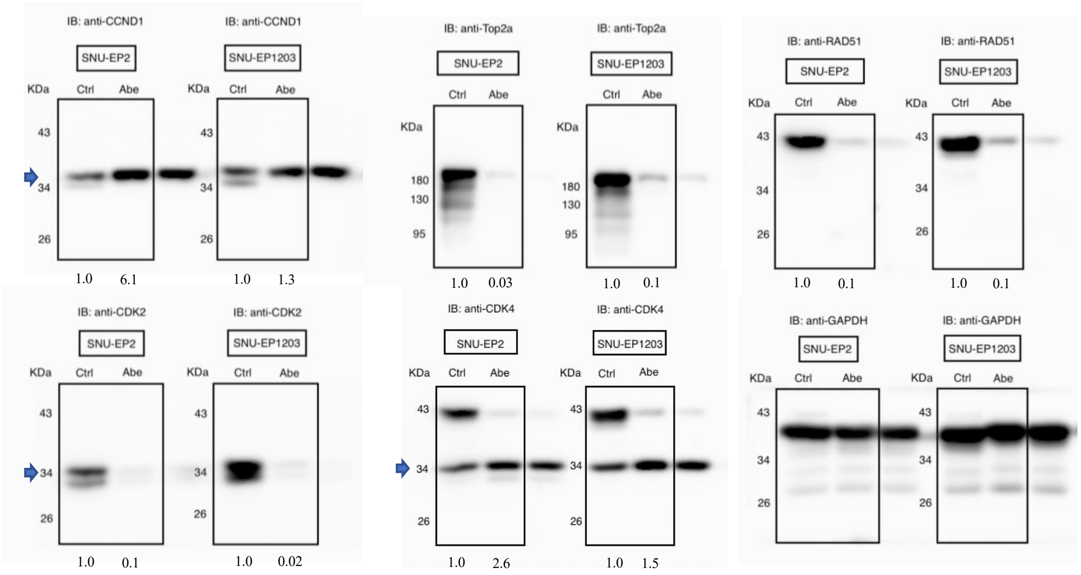

Figure 5C

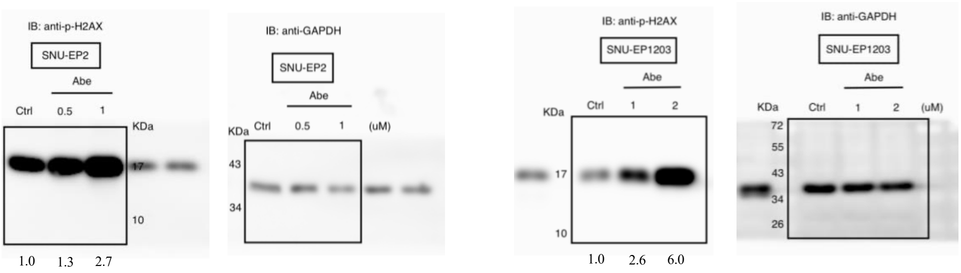

Figure 5D

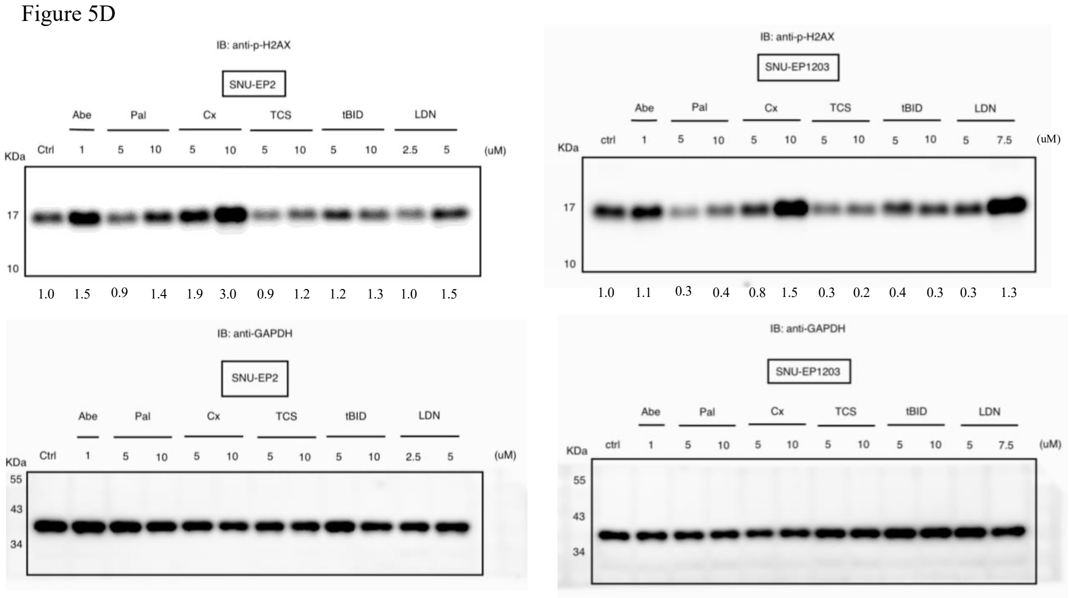

Figure S5. Uncropped Western blot images.
